# Acknowledgement to Reviewers of *Toxics* in 2017

**DOI:** 10.3390/toxics6010009

**Published:** 2018-01-17

**Authors:** Toxics Editorial Office

**Affiliations:** MDPI AG, St. Alban-Anlage 66, 4052 Basel, Switzerland

Peer review is an essential part in the publication process, ensuring that *Toxics* maintains high quality standards for its published papers. In 2017, a total of 42 papers were published in the journal. Thanks to the cooperation of our reviewers, the median time to first decision was 21 days and the median time to publication was 45 days. The editors would like to express their sincere gratitude to the following reviewers for their time and dedication in 2017:
Abballe, AnnalisaGoldoni, MatteoRainieri, SandraAgusa, TetsuroGreim, HelmutRegmi, BishnuAlaoui-Sosse, BadrGundert-Remy, UrsulaRice, LaShanta J.Almeida, Ana CatarinaGusiatin, MariuszRichardson, JasonAnadón, ArturoHaile, Tadele MeashoRietra, ReneAnderson, Stacey E.Halsall, CrispinRinge, DagmarAsimakopoulos, ΒyronHendryx, MichaelRobinson, Stacey A.Austin, RachelHöckner, MartinaRoca, MartaAvino, PasqualeHood, Darryl B.Roh, ChanghyunBailey, HelenHuang, Huang-ChiaoRoss, MatthewBarcelo, JuanHuertas-Pérez, José FernandoRubiano, Jesús GarcíaBend, JackHunt, PamelaRusso, Mario VincenzoBhaumik, AnusarkaHursthouse, AndrewSánchez, Miguel Ángel SogorbBoggaram, VijayIshaque, AliSanganyado, EdmondBonini, MarcelloKalinich, JohnSantilio, AngelaBurkholder, JamesKamo, MasashiSantos, Maria Teresa L. DosCappello, TizianaKilari, SreenivasuluSaraiva, NunoCarling, Gregory T.Kippler, MariaSas, ZoltanCaruso, Joseph A.Kojima, HiroyukiSchnell, IzhakCaserta, DonatellaKoneru, BhuvaneswariShea, Patrick J.Cavaleiro Rufo, JoãoKosters, AstridShinji, TokonamiCazzato, EugenioKusumbe, AnjaliSieber, NancyCesta, Mark F.Langley, Ricky L.Simon, EszterChalbot, Marie-Cecile G.Lavezzi, AnnaSpinazzè, AndreaChan, King MingLePrevost, Catherine E.Stacchiotti, AlessandraChi, Kai HsienLi, MilingStamatis, NikolaosChiang, Yi-TingLi, YangStark, John D.Chobot, VladimirLi, ZijianStefanelli, PatriziaClere, NicolasLoppi, StefanoSubramaniam, PrasadClift, MartinLuckenbach, TillSumi, DaigoColoso, ClaudioMartinez Illamola, SilviaSzyszkowicz, MieczyslawCorreia, ManuelaMavrogonatou, EleniTaira, JunseiCurl, Cynthia L.Melloni, EdonTao, LimingDailianis, StefanosMiller, CharlesTeschke, RolfDe Lourdes Pereira, MariaMoretto, AngeloTigini, ValeriaDe Oliveira Fernandes, EduardoMurata, YoshiyukiTsai, Wen-TienDesrosiers, MelanieNees, MatthiasVan Emon, Jeanette M.Doekes, GertNiemann, LarsViegas, SusanaDrasler, BarbaraNoller, BarryVioque, IgnacioDuodu, GodfredNurchi, Valeria M.Vliet, SaraEberhart, JohannOh, Jin-WooWallace, DavidEdwards, Joshua R.Parajuli, RajendraWalton, AnnMarieElholm, GPateraki, StylianiWang, ShengnianEngel, Paul C.Pelin, MarcoYang, XiaomeiEvelyn, TalbottPiña, BenjamiYiin, Lih-MingFaner, RosaPintado-Herrera, MarinaZamaratskaia, GaliaFasano, EvelinaPivonello, RosarioZara, SeverinoFavas, PauloPognonec, PhilippeZeiner, MichaelaFröhlich, EleonorePollitt, KrystalZhang, DayiGamble, Donald S.Poole, Jill A.Zhang, YingyueGamet-Payrastre, LaurencePrat, MariaZhang, ZhenhuaGarcía-Caballero, MelissaPuddu, Paolo EmilioGea, Oliveri ContiQuémerais, Bernadette

